# Pharmacist-Urologist Collaborative Management Improves Clinical Outcomes in Patients With Castration-Resistant Prostate Cancer Receiving Enzalutamide

**DOI:** 10.3389/fphar.2022.901099

**Published:** 2022-05-19

**Authors:** Masaki Hirabatake, Hiroaki Ikesue, Yuna Iwama, Kei Irie, Shintaro Yoshino, Toshinari Yamasaki, Tohru Hashida, Mutsushi Kawakita, Nobuyuki Muroi

**Affiliations:** ^1^ Department of Pharmacy, Kobe City Medical Center General Hospital, Kobe, Japan; ^2^ Department of Clinical Pharmacy, Faculty of Pharmaceutical Sciences, Kobe Gakuin University, Kobe, Japan; ^3^ Department of Urology, Kobe City Medical Center General Hospital, Kobe, Japan

**Keywords:** enzalutamide (ENZ), pharmacist, collaborative management, ambulatory care, Castration-Resistant Prostate Cancer (CRPC)

## Abstract

**Background:** Enzalutamide is useful for the treatment of castration-resistant prostate cancer (CRPC). Despite its usefulness, adverse events (AEs) sometimes force patients to discontinue treatment. To maximize patient care, we developed an ambulatory care pharmacy practice that allows collaboration between a pharmacist and urologist to manage patients with CRPC receiving enzalutamide. In this study, we investigated the efficacy of this collaborative management.

**Methods:** A retrospective chart review of 103 patients with CRPC receiving enzalutamide in our hospital between May 2014 and December 2020 was performed. Our collaborative management was implemented in October 2016. Before being examined by urologists, patients visited the oncology pharmacy consultation room for a face-to-face consultation, wherein the oncology pharmacists assessed factors such as adherence to enzalutamide, any AEs and their grades, and provided their suggestions to the urologists. The time to enzalutamide discontinuation and prostate-specific antigen progression were compared between patients who started enzalutamide before (*n* = 41) and after (*n* = 62) the implementation of the collaborative management. A multivariate Cox regression analysis was performed to analyze the factors associated with enzalutamide discontinuation.

**Results:** After implementing collaborative management, the pharmacists had 881 patient consultations. Among the 476 suggestions from pharmacists, 345 were accepted by urologists. The most frequent suggestion was supportive care in enzalutamide treatment (224 suggestions). Multivariate analysis showed that collaborative management [hazard ratio (HR) 0.53, 95% confidence interval (CI) 0.31–0.89, *p* = 0.017] and higher prostate-specific antigen (PSA; HR 2.41, 95% CI 1.36–4.28, *p* = 0.003) were significantly associated with enzalutamide discontinuation. The median time to discontinuation (18.9 vs. 7.6 months, *p* = 0.012), time to discontinuation due to AEs (not reached in both groups, *p* = 0.001), and time to PSA progression (13.3 vs. 5.8 months, *p* = 0.002) were all significantly longer in the after group.

**Conclusions:** We implemented a pharmacist-urologist collaborative management program for outpatients with CRPC receiving enzalutamide. The results revealed that collaborative management was useful for prolonging the time to enzalutamide discontinuation.

## Introduction

Prostate cancer (PC) is one of the most common malignancies worldwide. A total of 1,414,259 newly diagnosed cases of PC leading to 375,304 deaths were reported in 2020 (Sung et al., 2021). As PC is an androgen-dependent malignancy, androgen restriction therapy is commonly used as the first line of treatment (Cornford et al., 2021). Although androgen restriction therapy usually results in remission lasting 1–2 years, patients eventually develop castration-resistant prostate cancer (CRPC) due to a variety of factors (Wong et al., 2014). Docetaxel, introduced in 2004 for patients with CRPC, remains to be one of the important therapy options for these patients (Tannock et al., 2004). Even after the acquisition of a castration-resistant phenotype, the androgen receptor (AR) axis is a key element that promotes disease progression of PC (Wong et al., 2014; Rice et al., 2019).

Over the past decade, several new agents have been approved for the treatment of CRPC, including four androgen signaling inhibitors (ASIs): abiraterone acetate plus prednisone, apalutamide, darolutamide, and enzalutamide (Rice et al., 2019; Cornford et al., 2021; Schaeffer et al., 2021; Wei et al., 2021). Of these ASIs, enzalutamide was first approved in Japan in April 2014. Patients with CRPC who were treated with docetaxel and had disease progression showed a significantly longer time to progression and overall survival with enzalutamide (Scher et al., 2012), and chemotherapy-naïve patients showed a significantly longer time to progression and overall survival with enzalutamide (Beer et al., 2014; Hussain et al., 2018). Although enzalutamide proved to have better tolerance than that of docetaxel, with reduced incidences of hematologic toxicity and infectious disease, it has a different adverse event (AE) profile, including appetite loss, fatigue, skin toxicity, and hypertension (Scher et al., 2012; Beer et al., 2014; Hussain et al., 2018). The management of adverse effects was critical because these symptoms could cause patients to discontinue enzalutamide treatment (Joshua et al., 2015; Payne et al., 2021).

Studies have demonstrated that patient-centered, multidisciplinary team care, which provides support, information, and empowerment to cancer patients receiving chemotherapy, is effective (Pillay et al., 2016; Lutfiyya et al., 2019; Xie et al., 2021). In addition, we developed a pharmacist-physician collaborative management system for patients with idiopathic pulmonary fibrosis receiving pirfenidone treatment, and reported the efficacy of this approach (Satsuma et al., 2020). Based on these, in October 2016, we developed an ambulatory care pharmacy practice that allows pharmacist–urologist collaboration for the management of patients with CRPC receiving ASIs, including enzalutamide. Pharmacists meet and educate patients, assess AEs and adherence to enzalutamide in each patient, and suggest prescriptions to urologists based on patients’ conditions.

To date, few studies have investigated the efficacy of pharmacists in the treatment of patients with PC (Patel et al., 2016; Lankford et al., 2021). Lankford et al. evaluated pharmacist interventions regarding prescriptions for patients with cancer and reported that these interventions led to significant cost reduction in the total medical cost (Lankford et al., 2021). Patel et al. implemented a pharmacist-led oral chemotherapy monitoring program and evaluated its efficacy. Although the average number of interventions per patient and adherence to laboratory monitoring significantly increased after the implementation, the time to drug discontinuation was not prolonged (Patel et al., 2016).

In this study, we investigated the efficacy, including prolongation of time until enzalutamide discontinuation, of the pharmacist-urologist collaborative management for outpatients with CRPC receiving enzalutamide.

## Materials and Methods

### Implementing Pharmacist-Urologist Collaborative Management of Enzalutamide Treatment for Outpatients With PC

In October 2016, we launched an ambulatory care pharmacy practice that facilitated a pharmacist-urologist collaboration in the management of outpatients with PC receiving enzalutamide. The goals of our collaborative management were to assist urologists in prescribing appropriate medication to patients, managing AEs and drug-drug interactions, ensuring better adherence to therapy, and enhancing the efficacy of enzalutamide treatment. The ambulatory care pharmacy practice comprised three board-certified oncology pharmacists in Japan. The pharmacists had an average experience of 21.7 and 15.3 years in general and oncology practices, respectively. Before implementing collaborative management, that is, in conventional standard management, only urologists examined patients and prescribed enzalutamide. Medicine was then dispensed to the patients at community pharmacies. However, oncology pharmacists were not consulted.

**FIGURE 1:**
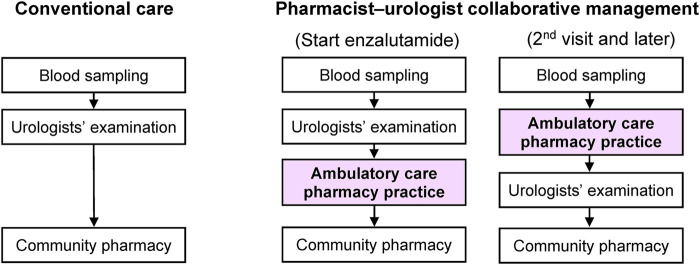
Flowchart of pharmacist-physician collaborative management and conventional care.

Figure 1 presents a patient flowchart that includes the ambulatory care pharmacy practice. During the first visit, urologists advised the patients to contact an ambulatory care pharmacy practice after their clinical examination when enzalutamide was first prescribed. Our ambulatory care pharmacy practice was conducted in a pharmacist consultation room, which is equivalent to physicians’ examination rooms and located in our hospital. Pharmacists record their consultation data into the electronic medical record (EMR) system in our hospital. An oncology pharmacist educated patients in a drug consultation room using a booklet on the following topics: 1) dosage and time of intake; 2) symptoms and management of enzalutamide-associated AEs; and 3) drug-drug interactions. To avoid drug-drug interactions, the oncology pharmacist checked the patients’ concomitant prescriptions and supplements.

During the second visit and later, before being examined by urologists, patients visited the oncology pharmacy consultation room for a 15 min face-to-face consultation, wherein the oncology pharmacists assessed: 1) adherence to enzalutamide by patient interview (patients’ self-report based on residual medication tablet counts); 2) any AEs and their grades according to the National Cancer Institute Common Terminology Criteria for Adverse Events (CTCAE) version 5.0; 3) whether the patient was able to take the medication for AEs appropriately; and 4) any changes in concomitant medications or supplements and provided their suggestions to the urologists mainly by reporting to the EMR system or *via* phone call if necessary. Oncology pharmacists entered their consultation records, including the aforementioned assessments and suggestions to the urologists, into the EMR system. Oncology pharmacists usually take around 5 min to enter their consultation records. After pharmacist consultation, patients visited the urologists’ examination room. Urologists examined and prescribed medication by referring to the pharmacist’s consultation records. The prescribed medication, including enzalutamide, was then dispensed at community pharmacies. The patient was regularly educated by the oncology pharmacist on how to manage AEs. The patient could ask the pharmacist any questions directly in the ambulatory care pharmacy practice or by telephone. Based on the assessment, the pharmacist suggested prescriptions to urologists as needed.

### Patients

**FIGURE 2:**
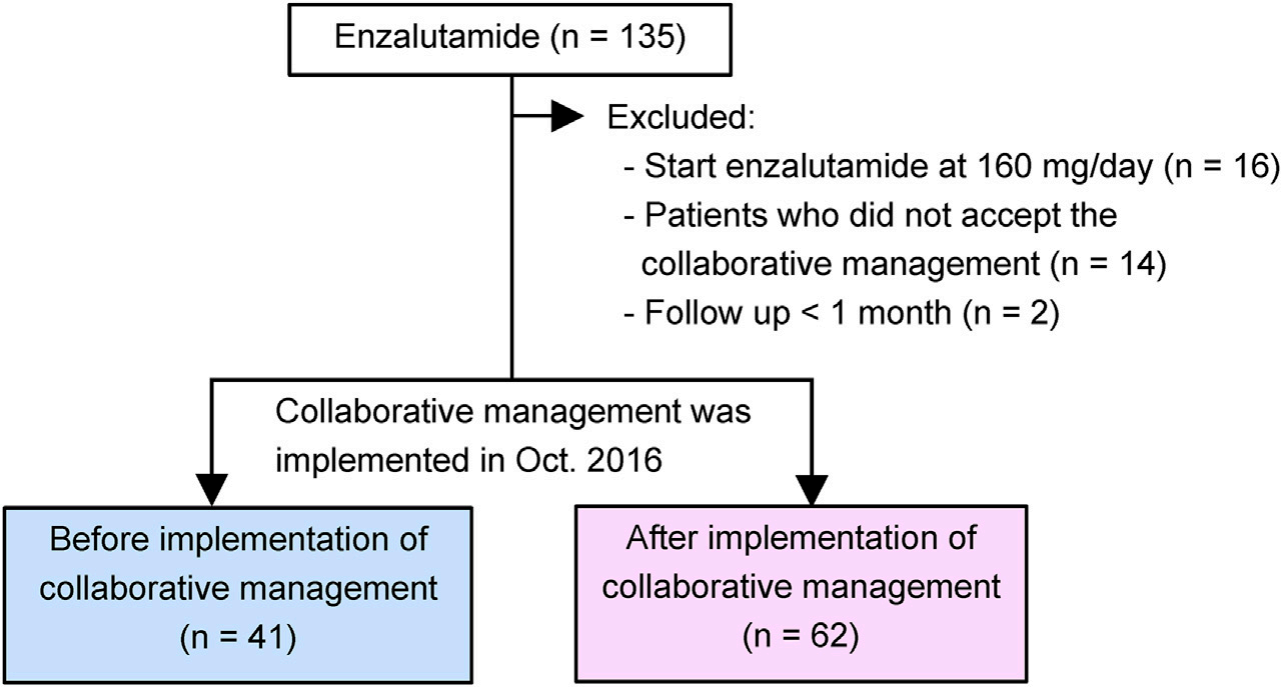
Study diagram. Our pharmacist-urologist collaborative management was implemented in October 2016. Patients who started enzalutamide until September 2016 were allocated to the before group (*n* = 41), and patients who started enzalutamide afterwards were placed in the after group (*n* = 62).

A retrospective chart review of 135 patients with CRPC was performed. The patients started enzalutamide treatment at the Department of Urology of the Kobe City Medical Center General Hospital from May 2014 to December 2020 (Figure 2). No patient was treated with abiraterone, enzalutamide, or taxanes before progression to CRPC. All study patients received enzalutamide once daily. Before January 2015, all patients received enzalutamide at 160 mg/day, and the dose was modified according to each patient’s condition. Thereafter, most patients started enzalutamide at 80–120 mg/day (initial dose reduction), and the dose was escalated to 120 and 160 mg sequentially while confirming tolerability (Vinh-Hung et al., 2020; Miura et al., 2021; Tsuzuki et al., 2021). Thus, we excluded 16 patients who were started enzalutamide at 160 mg/day in this study. We also excluded 14 patients who did not accept the collaborative management or two patients who could not be followed up for at least 1 month. As a result, 103 patients were included in this study. Forty-one patients were started on enzalutamide before implementing collaborative management (before group). After 1 October 2016, 62 patients began enzalutamide treatment (after group). This study was carried out in accordance with the Helsinki Declaration. The Kobe City Medical Center General Hospital Ethics Committee approved the protocol (approval number: zn210736).

### Data Collection and Outcomes

The primary endpoint was to investigate the efficacy of pharmacist-physician collaborative management for time to enzalutamide discontinuation. The secondary endpoints included time to enzalutamide discontinuation due to AEs, time to prostate-specific antigen (PSA) progression (an increase of 25% and an absolute increase of 2 ng/ml or more above the PSA nadir) (Scher et al., 2008), and suggestions provided to urologists by the oncology pharmacists in the collaborative management group. Time to enzalutamide discontinuation was defined as the time from initiation of enzalutamide to the date of treatment discontinuation for any reason. Time to enzalutamide discontinuation due to AEs was defined as the time from initiation of enzalutamide to the date of treatment discontinuation due to intolerable AEs. Time to PSA progression was defined as the time from initiation of enzalutamide to the date of PSA progression. All data were collected from the EMR system at our hospital. The upper limit of normal (ULN) levels of alkaline phosphatase (ALP) and lactate dehydrogenase (LDH) in our hospital were 340 U/L and 250 U/L, respectively. The data cut-off date was 31 October 2021.

### Statistics

Categorical variables are shown as the number of patients (n) and their frequencies. Chi-square tests or Fisher’s exact tests were used to compare the values. For continuous variables, median (interquartile range [IQR]) values are shown. The Wilcoxon rank-sum test was used to examine differences between the groups. To analyze factors associated with time to enzalutamide discontinuation or time to PSA progression, a Cox regression analysis was performed, utilizing patient’s age, PSA before starting enzalutamide, LDH, ALP, metastasis, Eastern Cooperative Oncology Group performance status (ECOG PS), previous treatments, duration of androgen deprivation therapy (ADT) before CRPC, hemoglobin, Gleason score, and before/after pharmacist-urologist collaborative management as independent variables. In the subsequent multivariable Cox regression analysis, significant factors in the univariate analyses were evaluated as potential covariates. The Kaplan-Meier method was used to estimate the time to events, and log-rank tests were used to compare the groups. At the last follow-up, patients who were still receiving enzalutamide and had not progressed, were censored. All statistical analyses were conducted using JMP version 13.2.1 (SAS Institute Inc, Cary, NC, United States), with a two-tailed *p*-value of <0.05, which was considered statistically significant.

## Results

### Baseline Characteristics of Patients

Between May 2014 and September 2016, 41 patients with CRPC started enzalutamide treatment before implementing the ambulatory care pharmacy practice (before group) (Figure 2). After implementing the ambulatory care pharmacy practice between October 2016 and December 2020, 62 patients with CRPC started enzalutamide treatment (after group).

The baseline patient characteristics are summarized in Table 1. The median (IQR) age was 77 years (70–84). Sixty-seven (65.7%) patients had metastatic CRPC and 64 (62.1%) had bone metastasis. The median (IQR) values of PSA and LDH at the start of enzalutamide treatment were 10.3 ng/ml (4.3–54.6) and 195 U/L (170–237), respectively. In the before and after groups, patient characteristics such as the median value of PSA at the start of enzalutamide (10.9 vs. 9.6 ng/ml, *p* = 0.547), and the median duration of ADT before CRPC (17.1 vs. 20.3 months, *p* = 0.806) were not significantly different. Only the proportion of previous abiraterone treatment (7.3 vs. 22.6%, *p* = 0.057) tended to be higher in the after group.

**TABLE 1:** Baseline patient characteristics.

Characteristics	Total (*n* = 103)	Implementation of Collaborative management		*p* value
		Before (*n* = 41)	After (*n* = 62)	
Age, years	77 (70–84)	77 (71–83)	77 (70–84)	0.941
Body weight, kg	60.6 (54.9–68.8)	59.3 (54.9–68.6)	62.2 (54.8–69.7)	0.330
ECOG PS, n (%)				
0	56 (54.4%)	20 (48.8%)	36 (58.1%)	0.475
1	34 (33.0%)	14 (34.2%)	20 (32.3%)	
≥2	13 (12.6%)	7 (17.1%)	6 (9.7%)	
Gleason score ≥8, n (%)	70 (68.0%)	26 (63.4%)	44 (71.0%)	0.518
PSA at start enzalutamide, ng/mL	10.3 (4.3–54.6)	10.9 (5.5–64.3)	9.6 (3.8–58.7)	0.547
Metastatic prostate disease, n (%)				
M0	35 (34.3%)	15 (36.6%)	20 (32.8%)	0.832
M1	67 (65.7%)	26 (63.4%)	41 (67.2%)	
Lymph node metastasis, n (%)	50 (48.5%)	20 (48.8%)	30 (48.4%)	1.000
Bone metastasis, n (%)	64 (62.1%)	26 (63.4%)	38 (61.3%)	1.000
Visceral metastasis, n (%)	14 (13.6%)	5 (12.2%)	9 (14.5%)	1.000
Hemoglobin, g/L	12.2 (10.4–13.3)	11.8 (10.3–13.2)	12.5 (10.8–13.4)	0.163
LDH, U/L	195 (170–237)	185 (167–241)	197 (172–237)	0.689
ALP, U/L	281 (205–414)	251 (197–389)	311 (225–426)	0.281
Duration of ADT before CRPC, months	18.4 (11.9–35.6)	17.1 (12.1–39.0)	20.3 (11.4–34.0)	0.806
<12 months, n (%)	26 (25.2%)	10 (24.4%)	16 (25.8%)	1.000
≥12 months, n (%)	77 (74.8%)	31 (75.6%)	46 (74.2%)	
Previous treatments, n (%)				
Taxanes	25 (24.3%)	11 (26.8%)	14 (22.6%)	0.645
Abiraterone acetate	17 (16.5%)	3 (7.3%)	14 (22.6%)	0.057

Continuous values are presented as the median (interquartile range [IQR]).

ADT, androgen deprivation therapy; ALP, alkaline phosphatase; CM, collaborative management; CRPC, castration-resistant prostate cancer; ECOG-PS, Eastern Cooperative Oncology Group performance status; LDH, lactate dehydrogenase; PSA, prostate-specific antigen.

### Effect of Pharmacist-Urologist Collaborative Management on Enzalutamide Treatment

**FIGURE 3:**
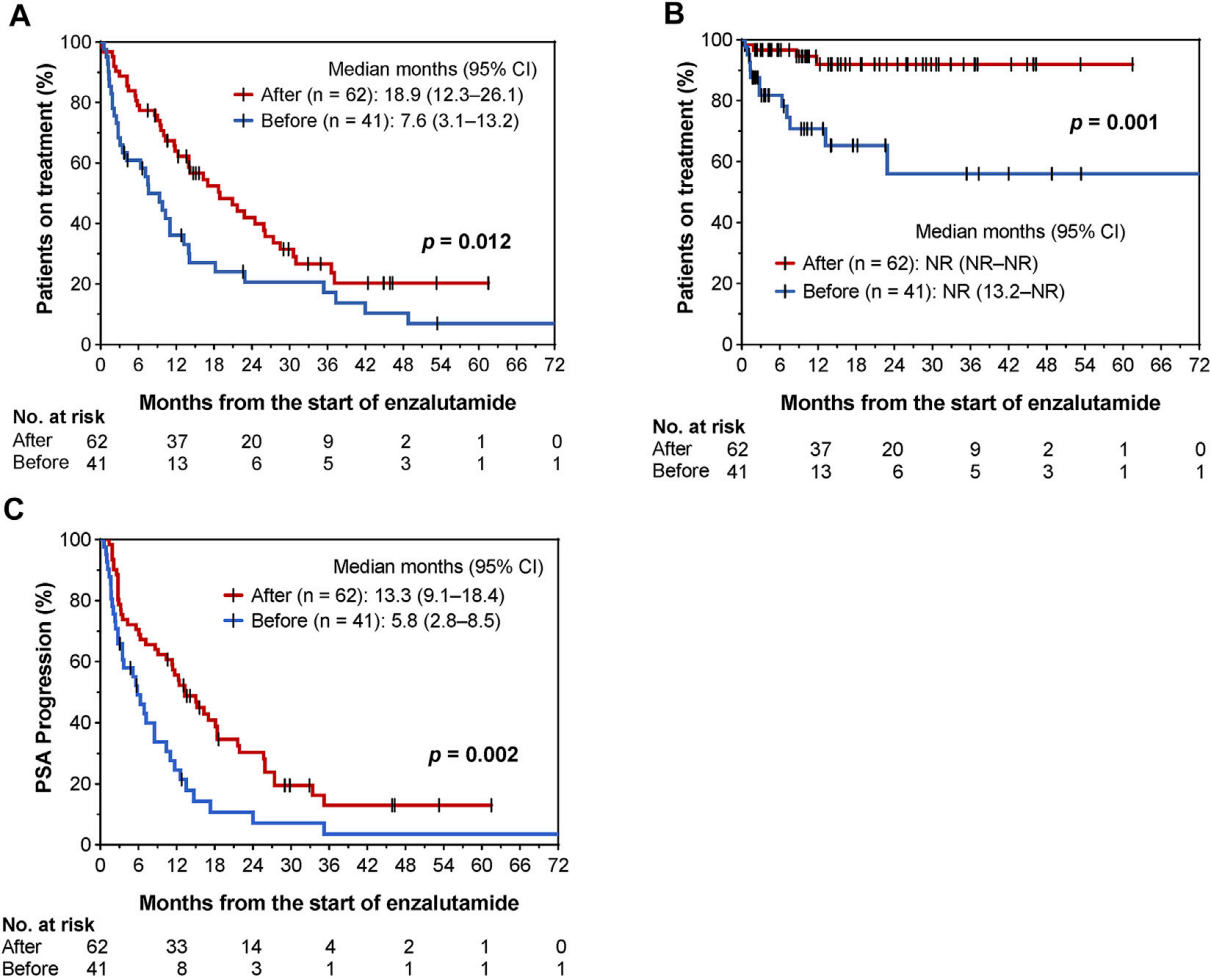
Kaplan–Meier curves for comparisons of time to enzalutamide discontinuation **(A)**, time to enzalutamide discontinuation due to AEs **(B)**, and time to PSA progression **(C)** before and after implementation of pharmacist-urologist collaborative management in patients receiving enzalutamide.

Until 31 October 2021, 76 and 81 patients discontinued enzalutamide and showed PSA progression, respectively. The Kaplan-Meier curves before and after the implementation of collaborative management are shown in Figure 3. The time to enzalutamide discontinuation [median 18.9 months (95% CI, 12.3–26.1) vs. 7.6 months (95% CI, 3.1–13.2); *p* = 0.012; Figure 3A], and the time to enzalutamide discontinuation due to AEs [median not reached (NR) (95% CI, NR–NR) vs. NR (95% CI, 13.2–NR); *p* = 0.001; Figure 3B] were both significantly longer in the after group than in the before group. The rates of enzalutamide discontinuation due to AEs were significantly decreased in the post-intervention group [29.3% (12/41) vs. 4.8% (3/62), *p* = 0.001]. The type and grade of AEs associated with enzalutamide discontinuation are shown in Supplementary Table S1. As a result, time to PSA progression was also prolonged in the after group [median 13.3 (95% CI, 9.1–18.4) vs. 5.8 months (95% CI, 2.8–8.5), *p* = 0.002; Figure 3C].

We used a Cox proportional hazards model to conduct univariate and multivariate analyses to assess the usefulness of the pharmacist-urologist collaborative management in the time to enzalutamide discontinuation. Collaborative management [hazard ratio (HR) 0.53, 95% confidence interval (CI) 0.31–0.89, *p* = 0.017], and PSA value at start enzalutamide > median (HR 2.41, 95% CI 1.36–4.28, *p* = 0.003) were significantly associated with enzalutamide discontinuation (Table 2). For time to PSA progression, collaborative management (HR 0.37, 95% CI 0.22–0.62, *p* < 0.001), PSA value at start enzalutamide > median (HR 2.58, 95% CI 1.41–4.76, *p* = 0.002), previous abiraterone treatment (HR 5.46, 95% CI 2.43–12.26, *p* < 0.001), and the duration of ADT before CRPC <12 months (HR 2.02, 95% CI 1.13–3.51, *p* = 0.018) were significantly associated (Table 3).

**TABLE 2:** Univariate and multivariate Cox proportional hazard models for time to enzalutamide discontinuation.

Variables	Univariate analyses		Multivariate analysis	
	Unadjusted HR (95% CI)	*p* value	Adjusted HR (95% CI)	*p* value
After implementation of collaborative management	0.56 (0.36–0.89)	0.015	0.53 (0.31–0.89)	0.017
PSA at start enzalutamide > median	3.77 (2.31–6.25)	<0.001	2.41 (1.36–4.28)	0.003
Duration of ADT before CRPC <12 months	2.11 (1.21–3.52)	0.009	1.67 (0.92–2.96)	0.092
Previous abiraterone treatment	3.15 (1.68–5.62)	0.001	1.73 (0.78–3.74)	0.176
Previous taxanes treatment	2.65 (1.56–4.38)	0.001	1.09 (0.59–1.94)	0.785
Metastatic disease	2.35 (1.43–4.01)	0.001	1.54 (0.87–2.76)	0.138
LDH at start enzalutamide > ULN	2.03 (1.07–3.59)	0.032	1.12 (0.55–2.15)	0.746
Hb at start enzalutamide <10 g/dl	1.83 (0.98–3.20)	0.058	N/A	N/A
Gleason score ≥8	1.44 (0.88–2.44)	0.151	N/A	N/A
ECOG PS ≥ 2	1.78 (0.85–3.32)	0.118	N/A	N/A
ALP at start enzalutamide > ULN	1.15 (0.70–1.84)	0.577	N/A	N/A
Age ≥75 years	0.78 (0.49–1.23)	0.317	N/A	N/A

ADT, androgen deprivation therapy; ALP, alkaline phosphatase; CI, confidence interval; CRPC, castration-resistant prostate cancer; ECOG-PS, Eastern Cooperative Oncology Group performance status; LDH, lactate dehydrogenase; HR, hazard ratio; PSA, prostate-specific antigen; ULN, upper limit of normal.

N/A indicates that the covariate was not included in the model because it was not significant in the univariate analysis.

**TABLE 3:** Univariate and multivariate Cox proportional hazard models for time to PSA progression from the initiation of enzalutamide.

Variables	Univariate analyses		Multivariate analysis	
	Unadjusted HR (95% CI)	*p* value	Adjusted HR (95% CI)	*p* value
After implementation of collaborative management	0.49 (0.31–0.78)	0.003	0.37 (0.22–0.62)	<0.001
PSA at start enzalutamide > median	4.07 (2.49–6.83)	<0.001	2.58 (1.41–4.76)	0.002
Duration of ADT before CRPC <12 months	2.12 (1.25–3.47)	0.006	2.02 (1.13–3.51)	0.018
Previous abiraterone treatment	7.26 (3.70–13.83)	<0.001	5.46 (2.43–12.26)	<0.001
Previous taxane treatment	3.58 (2.14–5.85)	<0.001	1.76 (0.86–3.40)	0.117
Metastatic disease	2.04 (1.27–3.37)	0.003	1.06 (0.61–1.87)	0.849
LDH at start enzalutamide > ULN	2.62 (1.40–4.56)	0.004	1.37 (0.68–2.59)	0.367
Hb at start enzalutamide <10 g/dl	2.76 (1.51–4.75)	0.001	1.30 (0.61–2.83)	0.501
Gleason score ≥8	1.66 (1.03–2.78)	0.038	1.21 (0.70–2.16)	0.506
ECOG PS ≥ 2	1.07 (0.44–2.20)	0.865	N/A	N/A
ALP at start enzalutamide > ULN	1.45 (0.90–2.29)	0.121	N/A	N/A
Age ≥75 years	0.92 (0.59–1.43)	0.701	N/A	N/A

ADT, androgen deprivation therapy; ALP, alkaline phosphatase; CI, confidence interval; CRPC, castration-resistant prostate cancer; ECOG-PS, Eastern Cooperative Oncology Group performance status; LDH, lactate dehydrogenase; HR, hazard ratio; PSA, prostate-specific antigen; ULN, upper limit of normal.

N/A indicates that the covariate was not included in the model because it was not significant in the univariate analyses.

### Activities of Oncology Pharmacists in the Ambulatory Care Pharmacy Practice

In the collaborative management, the oncology pharmacists had a total of 881 instances [median (IQR) 13 times (5–20 times)] of a face-to-face consultation with patients and provided 476 suggestions to urologists based on the enzalutamide treatment. Among these suggestions, the most frequent was supportive care in enzalutamide treatment (224 suggestions). The suggestions also included the enzalutamide dose (176 suggestions), laboratory tests (64 suggestions), and others (12 suggestions). As for suggestions for supportive care, gastrointestinal toxicities (91 suggestions), including constipation (47 suggestions) and anorexia (16 suggestions), were the most frequent, followed by skin toxicities (56 suggestions). Of the 476 suggestions from the oncology pharmacists, 345 (72.5%) were accepted by urologists and reflected in the prescriptions (Table 4).

**TABLE 4:** Number of suggestions provided by the pharmacists and urologists responses in 62 patients who were managed by the collaborative management.

	Number of suggestions	Number of suggestions accepted by urologists
Supportive care		
Gastrointestinal toxicity	91	81 (89.0%)
Skin toxicity	56	42 (75.0%)
Pain control	27	19 (70.4%)
Hypertension	26	20 (76.9%)
Fatigue	11	6 (54.5%)
Others	13	9 (69.2%)
Subtotal	224	177 (79.0%)
Enzalutamide dosage based on patients’ symptoms		
Increase the dosage	65	41 (63.1%)
Maintain the dosage	77	65 (84.4%)
Decrease the dosage	11	9 (81.8%)
Adjustment of the prescription days due to residue of enzalutamide tablets	12	5 (41.7%)
Others	11	8 (72.7%)
Subtotal	176	128 (72.7%)
Laboratory tests	64	33 (51.6%)
Others	12	7 (58.3%)
TOTAL	476	345 (72.5%)

## Discussion

We implemented and evaluated an ambulatory care pharmacy practice for pharmacist-urologist collaborative management of outpatients with CRPC receiving enzalutamide treatment. The rates of enzalutamide discontinuation due to AEs significantly decreased after the implementation of the ambulatory care pharmacy practice; the time to drug discontinuation, time to drug discontinuation due to AEs, and time to PSA progression were significantly prolonged after the implementation of collaborative management. Furthermore, a multivariate analysis clearly revealed that collaborative management significantly reduced the risk of enzalutamide discontinuation. To our knowledge, this is the first study to show that collaborative management of patients with prostate cancer by pharmacists and urologists in ambulatory care successfully prevents treatment discontinuation.

The value of pharmacist-led team care in pharmacotherapies has been demonstrated in numerous studies (Yamamoto et al., 2018; Escudero-Vilaplana et al., 2020; Satsuma et al., 2020; Ji et al., 2021; Jia et al., 2021; Marzal-Alfaro et al., 2021; Miura et al., 2021; Xu et al., 2021). The results of this study revealed that pharmacist-urologist collaborative management can also improve care for patients with CRPC receiving enzalutamide in an ambulatory care setting. A decrease in adherence due to AEs and a patient’s lack of understanding can also affect survival outcomes (Behl et al., 2017). In our ambulatory care pharmacy practice, pharmacists played several important roles: interviewing and educating patients, suggesting appropriate supportive care, suggesting enzalutamide dose adjustments to physicians if necessary, managing drug-drug interactions, and encouraging patients to adhere to enzalutamide. The majority of suggestions made by the pharmacists were supportive care medicines (224/476 suggestions), followed by a dose of enzalutamide (176 suggestions). Of the 476 suggestions, urologists accepted most cases (72.5%). Gastrointestinal toxicities including constipation (30 suggestions) and anorexia (16 suggestions) were the most frequent suggestions among supportive care. Pharmacists have suggested dose adjustment of enzalutamide based on the severity of fatigue, because some studies suggest this as a potential approach to managing this symptom (Terada et al., 2016; Vinh-Hung et al., 2020; Miura et al., 2021; Tsuzuki et al., 2021). The various interventions by pharmacists seemed to contribute to prolonging the time to enzalutamide discontinuation and time to PSA progression. In addition, since enzalutamide has many drug-drug interactions, we often provide drug information to urologists. For example, in patients taking warfarin, we suggested that urologists measure the prothrombin time-international normalized ratio (PT-INR) and adjust the warfarin dose based on the PT-INR value.

On contrary, we speculated that evidence-based suggestions are more likely to be accepted by urologists, while suggestions with insufficient clinical evidence are less likely to be accepted. According to supportive care, pharmacists often suggest topical moisturizers and Chinese herbal medicines for mild skin rash and fatigue, respectively. The efficacy of those medications for enzalutamide-associated symptoms has not been validated. In suggestions according to enzalutamide dosage, pharmacists usually consider dose escalation every 2 weeks while confirming tolerability (Miura et al., 2021). However, urologists often make more careful decisions, especially in elderly patients.

The time to PSA progression varies according to patient characteristics. In phase Ⅲ randomized controlled trials, these times were 11.2, 8.3, and 37.2 months in chemotherapy naïve patients with metastatic CRPC, chemotherapy-treated patients with metastatic CRPC, and chemotherapy naïve patients with non-metastatic CRPC, respectively (Scher et al., 2012; Beer et al., 2014; Hussain et al., 2018). In real-world observational studies, the times to PSA progression were 2.3–27.0 months, which differed more from the patient populations of the studies (Badrising et al., 2014; Cheng et al., 2015; Terada et al., 2016; Smith et al., 2017; de Bono et al., 2018; Emamekhoo et al., 2018; Fujiwara et al., 2020; Jung et al., 2020; Yokomizo et al., 2022). In our study, the times to PSA progression were significantly improved from 5.8 to 13.3 months before and after the implementation of collaborative management groups, respectively, and both of which were within the range of those in previous studies.

Other factors, such as higher PSA at the start of enzalutamide, shorter duration of ADT before CRPC, and previous treatments were significantly associated with the time to enzalutamide discontinuation. These results are consistent with those of previous studies (Emamekhoo et al., 2018; Terada et al., 2016; Tsuzuki et al., 2021; Yokomizo et al., 2022).

Our study has a few limitations. First, it was a non-randomized, single-center observational study with retrospective evaluation of outcomes, such as time to enzalutamide in historical controls. Our preliminary results need to be confirmed by further prospective investigations at other institutions. Second, the oncology pharmacists played several important roles: educating patients, suggesting appropriate supportive care, suggesting enzalutamide dose adjustments to physicians if necessary, and encouraging patients to adhere to enzalutamide. The design of this study did not clarify the type of involvement of oncology pharmacists that contributed to improving the outcomes. Third, we excluded patients who started enzalutamide at a dose of 160 mg/day. If we analyze data which including the patients who started enzalutamide at 160 mg/day, the time to enzalutamide discontinuation and time to PSA progression were still significantly prolonged in the collaborative management group (data not shown). Several studies have reported that this factor had no effect on the time to enzalutamide discontinuation or PSA progression (Terada et al., 2016; Vinh-Hung et al., 2020; Miura et al., 2021; Tsuzuki et al., 2021). On contrary, one retrospective study suggested inferior oncological outcomes when treated with reduced-dose androgen receptor pathway inhibitors for CRPC (Yamada et al., 2022). The study also suggested that full-dose administration of those medications for CRPC may be appropriate if feasible. The effect of dose reduction in enzalutamide treatment needs further investigation. In addition, this study excluded the patients who did not accept the collaborative management. The starting dose of enzalutamide has changed during this study. The primary endpoint in this study was time to enzalutamide discontinuation, which was affected by efficacy and toxicity. Moreover, the physician’s assessment could modify this endpoint. Thus, these potential biases need to be addressed.

In conclusion, we developed an ambulatory care pharmacy practice for pharmacist-urologist collaborative management of outpatients with CRPC receiving enzalutamide. The findings suggest that, when compared to conventional management, collaborative management is effective at prolonging the time to enzalutamide discontinuation and time to PSA progression.

## Data Availability

The original contributions presented in the study are included in the article/Supplementary Material, further inquiries can be directed to the corresponding author.

## Abbreviations

AE, adverse event; CI, confidence interval; NR, not reached; PSA, prostate-specific antigen.
