# Supplementary material for: Pharmacist-Urologist Collaborative Management Improves Clinical Outcomes in Patients With Castration-Resistant Prostate Cancer Receiving Enzalutamide
Source: Front Pharmacol. 2022 May 19;13:901099. doi: 10.3389/fphar.2022.901099 (PMC9162505; doi:10.3389/fphar.2022.901099)
Supplement: Supplementary file 1 [file Table1.docx]

**Supplementary Table 1. Type and grade of adverse events associated with enzalutamide discontinuation**

| Type of AEs (grades) | No. of patients |
| --- | --- |
|  |  |
| Fatigue (grade 3) | 3 |
| Fatigue (grade 2) | 4 |
| Anorexia (grade 2) | 6 |
| Skin rash (grade 3) | 1 |
| ALT (grade 2) and AST (grade 1) elevation | 1 |
| Exacerbation of Parkinson's disease which he had at baseline (NA) | 1 |

AEs, adverse events; ALT, alanine aminotransferase; AST, aspartate aminotransferase.

N/A indicates that the grade of event was not applicable.
